# Label-Free Electrochemical Detection of Vanillin through Low-Defect Graphene Electrodes Modified with Au Nanoparticles

**DOI:** 10.3390/ma11040489

**Published:** 2018-03-25

**Authors:** Jingyao Gao, Qilong Yuan, Chen Ye, Pei Guo, Shiyu Du, Guosong Lai, Aimin Yu, Nan Jiang, Li Fu, Cheng-Te Lin, Kuan W.A. Chee

**Affiliations:** 1 Key Laboratory of Marine Materials and Related Technologies, Zhejiang Key Laboratory of Marine Materials and Protective Technologies, Ningbo Institute of Materials Technology and Engineering (NIMTE), Chinese Academy of Sciences, Ningbo 315201, China; gaojingyao@nimte.ac.cn (J.G.); Qilong.Yuan@nottingham.edu.cn (Q.Y.); yechen@nimte.ac.cn (C.Y.); guopei@nimte.ac.cn (P.G.); 2 College of Material Science and Optoelectronic Technology, University of Chinese Academy of Sciences, 19 A Yuquan Rd., Shijingshan District, Beijing 100049, China; 3 Department of Electrical and Electronic Engineering, Faculty of Science and Engineering, University of Nottingham, Ningbo 315100, China; Kuan.Chee@nottingham.edu.cn; 4 Department of Physics, Liaoning University, Shenyang 110000, China; 5 Ningbo Institute of Materials Technology and Engineering, Chinese Academy of Sciences, Ningbo 315201, China; dushiyu@nimte.ac.cn; 6 Department of Chemistry, Hubei Normal University, Huangshi 435002, China; gslai@hbnu.edu.cn; 7 Department of Chemistry and Biotechnology, Faculty of Science, Engineering and Technology, Swinburne University of Technology, Hawthorn, VIC 3122, Australia; aiminyu@swin.edu.au; 8 College of Materials and Environmental Engineering, Hangzhou Dianzi University, Hangzhou 310018, China

**Keywords:** low-defect graphene, sp^3^-to-sp^2^ conversion, gold nanoparticles modification, vanillin, electrochemical detection

## Abstract

Graphene is an excellent modifier for the surface modification of electrochemical electrodes due to its exceptional physical properties and, for the development of graphene-based chemical and biosensors, is usually coated on glassy carbon electrodes (GCEs) via drop casting. However, the ease of aggregation and high defect content of reduced graphene oxides degrade the electrical properties. Here, we fabricated low-defect graphene electrodes by catalytically thermal treatment of HPHT diamond substrate, followed by the electrodeposition of Au nanoparticles (AuNPs) with an average size of ≈60 nm on the electrode surface using cyclic voltammetry. The Au nanoparticle-decorated graphene electrodes show a wide linear response range to vanillin from 0.2 to 40 µM with a low limit of detection of 10 nM. This work demonstrates the potential applications of graphene-based hybrid electrodes for highly sensitive chemical detection.

## 1. Introduction

Vanillin (4-hydroxy-3-methoxybenzaldehyde) is the flavor component of vanilla bean, which is widely used in foods, beverages, and medicines [1,2], because it has an attractive aroma, and favorable antioxidative and antimicrobial properties [3,4,5]. However, excessive intake of vanillin will lead to headaches, nausea, vomiting, and even liver and kidney problems [6,7,8]. As a result, it is essential to develop a fast and reliable technique for vanillin determination especially for pharmaceutical and food analysis. Some methods have been used to detecting vanillin, such as surface-enhanced infrared absorption spectroscopy [9], surface plasmon resonances [9], micellar electrokinetic chromatography [10], high-performance liquid chromatography [11], and electroanalysis [12,13,14]. Among these methods, electroanalysis is most appreciated because of its fast detection response and high sensitivity.

Graphene is a two-dimensional material composed of hexagonally constructed sp^2^-bonded carbon atoms. It has extensive influence in the field of electrochemistry due to its exceptional electrical properties, like high carrier mobility (≈15,000 cm^2^/(V·s)) and high specific surface area (2600 m^2^/g) [15,16,17,18,19]. Graphene-based electrochemical sensors have been widely applied in electroanalysis and are usually fabricated by drop casting reduced graphene oxide (rGO) on the surface of glassy carbon electrodes (GCEs) [20,21,22,23]. However, the ease of aggregation caused by the van der Waals forces between adjacent graphene sheets will lead to the reduction in specific surface area and poor solubility in water [24]. Moreover, the introduction of oxygen-containing functional groups on the rGO surface gives rise to high intrinsic electrical resistance [25].

Compared to rGO, chemical-vapour-deposited (CVD) graphene exhibits lower electrical resistance and fewer structural defects. However, the lack of functional groups on the surface of CVD graphene limits its electrochemical activity. Therefore, it is efficient to improve the device sensitivity based on CVD graphene via further surface modification, such as molecular doping, the grafting of functional groups, and the decoration of noble metal nanoparticles [26,27,28]. Recently, Au nanoparticles (AuNPs) have attracted considerable attention owing to their unique electrocatalytic activity as well as excellent conducting capability [29,30,31,32]. When AuNPs were decorated on a graphene surface, they worked as electroactive sites for electrochemical reaction and enhanced the electron transfer efficiency of the graphene [32]. This synergistic effect on sensing applications makes AuNP/graphene hybrids more sensitive in chemical sensor applications.

In this work, we fabricated graphene-diamond hybrid electrodes by a catalytic annealing process using a thin Ni layer as catalyst. High-quality graphene films were formed directly on the diamond surface through an in-situ sp^3^-to-sp^2^ conversion process. Then, AuNPs were electrodeposited on the graphene surface using a simple cyclic voltammetry (CV) method. The fabricated electrode exhibits a linear electrochemical detection range of 0.2–4 µM with a low detection limit of 10 nM, demonstrating potential applications of graphene-based hybrid electrodes for highly sensitive chemical detection.

## 2. Experimental

### 2.1. Materials

High-pressure and high-temperature (HPHT) diamonds with dimensions of 3.5 × 3.5 × 1 mm were purchased from Shenzhen Tiantian Xiangshang Diamond Co. Ltd., Shenzhen, China. Nickel (99.995%) was brought from New Material Technology Co. Ltd., Beijing, China. H_2_O_2_, H_2_SO_4_, CuSO_4_, KCl, HCl, Na_2_HPO_4_, NaH_2_PO_4_, and vanillin were purchased from Sinopharm Chemical Reagent Co. Ltd. Shanghai, China.

### 2.2. Sample Preparation

The HPHT diamond substrate used in this study was comprised of (1 0 0) singe-crystal diamond. The diamond substrate was first immersed in a mixture of sulfuric acid and hydrogen peroxide (H_2_SO_4_:H_2_O_2_ = 7 mL:3 mL) solution at 50 °C for 4 h to remove surface contaminants, followed by ultrasonic cleaning in deionised water and ethanol for 10 min, respectively. A 20-nm-thick Ni film was deposited on the diamond surface via an e-beam system (MUE-ECO, Chigasaki, Japan) at a base pressure and deposition rate of 1.4 × 10^−5^ Torr and 0.5 Å/s, respectively. The Ni/diamond substrate was then thermally treated at 1020 °C for 15 min in a tube furnace system (BTF-1200C-II-SL, Hefei, China), with H_2_ flow of 8 sccm. Finally, the graphene–diamond hybrid electrodes were obtained after Ni removal in an etching solution (10 g CuSO_4_ and 50 mL HCl in 50 mL deionised water). The graphene–diamond hybrid sample was immobilised on a plastic substrate and connected by silver paints to silver wire. Except for the graphene surface, other exposed areas were protected by silicone resin. Au nanoparticles were then electrodeposited on the graphene surface via CV at a scan rate of 100 mV/s with 0.2 mL of 1% AuCl_4_ solution diluted in 10 mL PBS (PH = 7.0) solution with deposition times varying from 2 to 16 min.

### 2.3. Characterisations

The quality of the HPHT diamond and the obtained graphene were characterised by Raman spectroscopy with a 532 nm exciting wavelength of an He–Ne laser (Renishaw inVia Reflex, Renishaw plc, Wotton-under-Edge, UK) and X-ray photoelectron spectroscopy (XPS AXIS ULTR DLD, Kratos Analytical, Manchester, UK). The surface morphology was observed using a field emission scanning electron microscope (FE-SEM QUANTA 250 FEG, FEI, Hillsboro, OR, USA). The electrochemical experiments were carried out with an Autolab workstation (PGSTAT 302F, Metrohm, Herisau, Switzerland). The mercurous chloride reference and Pt counter electrode were obtained from Aida Hengsheng Co. Ltd., Tianjin, China.

## 3. Results and Discussion

**Figure 1:**
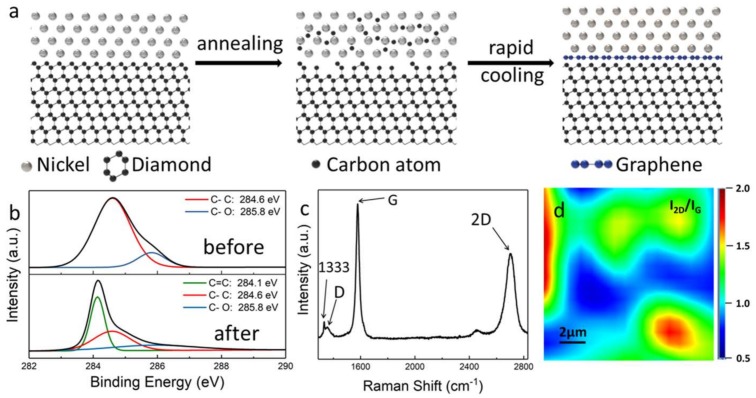
(**a**) Schematic illustration of the conversion process of the graphene–diamond hybrid through catalytic thermal treatment. (**b**) XPS C1s spectrum of pristine HPHT diamond before and after sp^3^-to-sp^2^ conversion. (**c**) A typical Raman spectrum and (**d**) Raman mapping of graphene films formed on the diamond surface.

As schematically illustrated in Figure 1a, first, the high-quality graphene on the diamond was prepared via catalytically thermal treatment based on the sp^3^-to-sp^2^ conversion process, in which the HPHT diamond was used as a carbon source. The sp^3^-bonded carbon atoms on the diamond surface were dissolved into a thin nickel film (≈20 nm) at high temperature (1020 °C) and then precipitated from the nickel film to form sp^2^-bonded carbons during a rapid cooling process. The success of sp^3^-to-sp^2^ conversion can be demonstrated by XPS analysis, which was used to determine the bonding information of the diamond surface before and after catalytic thermal treatment. As shown in Figure 1b, the pristine diamond was composed of complete sp^3^ bonds (C–C), while the ratio of sp^2^–sp^3^ bonds of the graphene–diamond surface increased to 1:1 after annealing. The quality of the obtained graphene was investigated with the Raman spectrometer, as shown in Figure 1c, in which the peaks located at 1350 cm^−1^, 1586 cm^−1^, and 2698 cm^−1^ can be assigned to the D-band, G-band, and 2D-band of graphene, respectively. The I_2D_/I_G_ ratio (≈0.62) and full width at half maximum (FWHM) of the 2D-band (≈64.5 cm^−1^) suggest that the graphene is composed of few layers [33]. In addition, the significantly low I_D_/I_G_ ratio (<0.1) reflects that the graphene converted from diamond has low defect content [33]. We noticed that there was a weak peak at 1333 cm^−1^, which can be assigned to the single-crystal diamond [34], confirming the nanometer-thick nature of graphene formed on the diamond surface. The Raman mapping in Figure 1d demonstrates that the I_2D_/I_G_ ratio of graphene film mostly varied between 1.0 and 1.5, showing that the diamond surface was covered by few layers of graphene [33].

**Figure 2:**
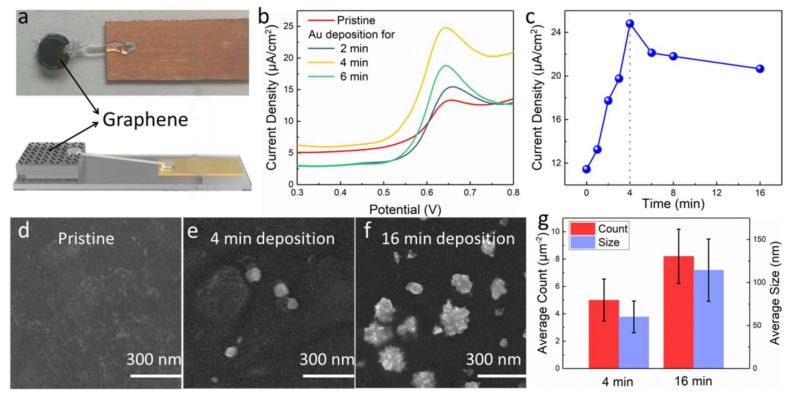
(**a**) Photograph of as-prepared graphene-diamond hybrid electrode. (**b**) CV responses of the electrodes with different deposition periods of AuNPs in PBS containing 50 µM vanillin (scan rate: 100 mV/s). (**c**) The changes of peak current density of prepared electrodes with different deposition periods. SEM images of (**d**) pristine graphene and AuNP/graphene after (**e**) 4 and (**f**) 16 min of electrodeposition. (**g**) The average count and size of AuNPs.

In order to enhance the sensitivity of graphene hybrid electrodes for detecting vanillin, the as-prepared electrode was further modified with AuNPs via electrodeposition. Figure 2a is a photograph of the as-prepared electrode. CV curves of the hybrid electrodes at various deposition periods in a 50 µM vanillin PBS solution were studied. As shown in Figure 2b, all electrodes display an oxidation peak at around 0.64 V, corresponding to the oxidation reaction of vanillin. Compared to the pristine electrode, the peak current density of modified electrodes presents a remarkable electrocatalytic enhancement. The peak current density of the electrodes after 4 min of deposition shows a two-fold improvement of the pristine one, suggesting that the decoration of AuNPs could highly promote the electro-oxidation of vanillin. Moreover, as presented in Figure 2c, the peak current density of the modified electrode increases first and then decreases as the deposition time is longer than 4 min. This can be attributed to the morphological change of AuNPs on the graphene surface with the increase in deposition time, as shown in Figure 2d–g. Compared to the pristine electrodes (Figure 2d), AuNPs can be observed on all the modified electrodes. The average count of AuNPs on the graphene surface is ≈5 μm^−2^ and ≈8 μm^−2^ for the samples after electrodeposition for 4 and 16 min, respectively, as exhibited in Figure 2e,f. In addition, it can be seen that the average size of AuNPs becomes larger (from 60 to 115 nm) with the increase in deposition time (from 4 to 16 min). Accordingly, the variation of the current density presented in Figure 2c can be explained as follows: Initially, with the increase in AuNP density, the electro-oxidation of vanillin was enhanced due to the increase in electrochemical reaction site, leading to the rise in peak current density. However, the size of AuNPs becomes larger when the deposition time is longer than 4 min, resulting in the decline of electrochemical performance of AuNPs [35] and the decrease in electro-oxidation reaction of vanillin.

**Figure 3:**
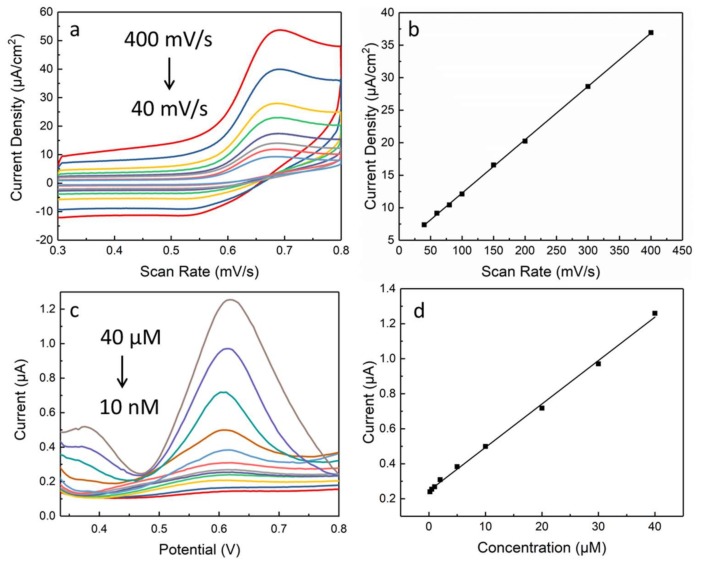
(**a**) CV responses of AuNP/graphene electrodes after 4-min electrodeposition in 50 μM vanillin/PBS buffer with various scan rates and (**b**) the corresponding current density of oxidation peak. (**c**) DPV curves of AuNP/graphene electrodes with different vanillin concentrations and (**d**) the linear dynamic detection range.

The mechanism of AuNP-decorated graphene electrodes for vanillin detection was investigated by changing the scan rate of CV measurement. As shown in Figure 3a, the peak current increases, and oxidation peak potential shifts to the positive direction as scan rate increases from 40 to 400 mV/s. The current density of oxidation peak as a function of the scan rate is shown in Figure 3b, which shows a good linear relationship with the correlation coefficient value of 0.999, indicating that the oxidation of vanillin on the proposed electrodes is a typical adsorption-controlled process [22]. The electrochemical method of differential pulse voltammetry (DPV) was applied to determine the low detection limit of vanillin. A typical DPV response carried out in PBS buffer (pH = 7) for different concentrations of vanillin (from 10 nM to 40 µM) can be seen in Figure 3c. It is obvious that the peak current of vanillin with the potential around 0.6 V increases as the concentration of vanillin rises from 10 nM to 40 µM. As shown in Figure 3d, the peak current shows a linear relation as a function of vanillin concentration, with the equation expressed as $I_{P}\left(\mu A\right)=0.245+0.025c\;\left(\mu M\right)$ and the correlation coefficient as 0.999 (from 0.2 µM to 40 µM). This good linear relationship confirmed that the sample after 4 min of electrodeposition of AuNPs can be successfully used for the detection of vanillin. The low limit of detection was demonstrated at about 10 nM in this work, showing higher sensitivity and a lower detection limit compared to reported works [13,21]. Our findings prove that the formation of AuNP/high-quality graphene hybrids enables higher electrochemical sensitivity than does that of electrodes prepared by drop casting rGO.

## 4. Conclusions

Based on an sp^3^-to-sp^2^ conversion process, the graphene–diamond hybrid electrodes were fabricated via catalytic thermal treatment. The low-defect graphene electrodes were further modified by electrodeposition of AuNPs using the CV method, and the deposition time was optimised to 4 min for achieving a good trade-off between the size (≈60 nm) and surface density of AuNPs, resulting in the highest electrochemical sensitivity for vanillin detection. As a result, a wide linear dynamic range of the hybrid electrodes can be achieved from 0.2 to 40 µM with a very low limit of detection of 10 nM. The AuNP/graphene sensing platform is highly efficient for vanillin determination in food, cosmetic, and pharmaceutical analysis.
